# Cannabidiol corrects sleep deficits and reduces spontaneous seizures in Angelman syndrome model mice

**DOI:** 10.1038/s41386-026-02462-7

**Published:** 2026-06-09

**Authors:** Tyler Shannon, Mariam Najeeb, Yoon-Jae Yi, Yuyan Shen, Bin Gu

**Affiliations:** 1 Department of Neuroscience, The Ohio State University, Columbus, OH USA; 2 Neuroscience Program, The Ohio State University, Columbus, OH USA; 3 College of Veterinary Medicine, The Ohio State University, Columbus, OH USA

**Keywords:** Preclinical research, Autism spectrum disorders

## Abstract

The off-label use of cannabidiol (CBD) has outpaced investigation. We assessed the effects of CBD in Angelman syndrome model mice lacking the *Ube3a* gene and found that chronic injection of CBD increased rapid-eye movement sleep during the dark cycle, restored “Siesta”, improved sleep homeostasis, and reduced spontaneous seizures following flurothyl kindling.

## Introduction

Cannabidiol (CBD), a major non-psychoactive and non-psychotomimetic phytocannabinoid constituent of cannabis, is gaining attention for its medical benefits. The multitarget and complex pharmacological nature of CBD in the central nervous system makes it a unique candidate for treating a wide range of complex neurological and psychiatric conditions [1], including seizures and sleep deficits. Though Epidiolex^®^, a pharmaceutical formulation of CBD, has been approved for treating three rare epilepsy conditions (Dravet syndrome, Lennox-Gastaut syndrome, and tuberous sclerosis complex), growing interest in and off-label medical use of CBD have outpaced preclinical and clinical research [2]. Angelman syndrome (AS) is a severe genomic imprinting disorder caused by loss of function of the maternally inherited *UBE3A* (Ubiquitin-ligase protein E3A) gene. Individuals with AS have a wide spectrum of neurological symptoms, including intellectual disability, motor dysfunction, sleep disturbances, and epilepsy [3]. No effective therapies exist for AS. AS model mice lacking the maternal *Ube3a* gene (*Ube3a*^*m–/p+*^) exhibit many core pathologies of AS, including behavioral deficits, sleep disturbances, EEG abnormalities, and enhanced susceptibility to convulsive stimuli [4]. Together, these characteristics make AS model mice a valuable preclinical model for evaluating the effects of CBD on seizures and sleep deficits [5]. Our previous study indicates that acute treatment of CBD attenuated the severity of audiogenic and hyperthermia-induced seizure and normalized delta (0.5–4 Hz) rhythms in AS model mice [6]. Notwithstanding recent efforts, two knowledge gaps still exist that halt the rational development of CBD as a therapy for AS: (1) Can CBD ameliorate sleep abnormalities in AS? (2) Can CBD reduce spontaneous seizures, the hallmark of epilepsy, in AS? Rigorous preclinical investigation of CBD in AS model mice will guide the rational development of CBD-based therapies for AS and ultimately improve the quality of life of affected individuals and their caregivers.

## Materials and methods

All animal procedures were approved by the Institutional Animal Care and Use Committee of The Ohio State University and were performed under the guidelines of the U.S. National Institutes of Health. Detailed information on AS (*Ube3a*^*m–/p+*^) model mice and their WT (*Ube3a*^*m+/p+*^) littermates (Jackson Laboratory; B6.129S7-*Ube3a*^*tm1Alb*^/J), treatment, surgery, video-EEG-EMG recordings, sleep analyses, power spectral analyses, flurothyl kindling, spontaneous seizure analysis, and statistics can be found in the Supplemental Materials.

## Results

### CBD corrects sleep deficits in naïve AS model mice

**Fig. 1:**
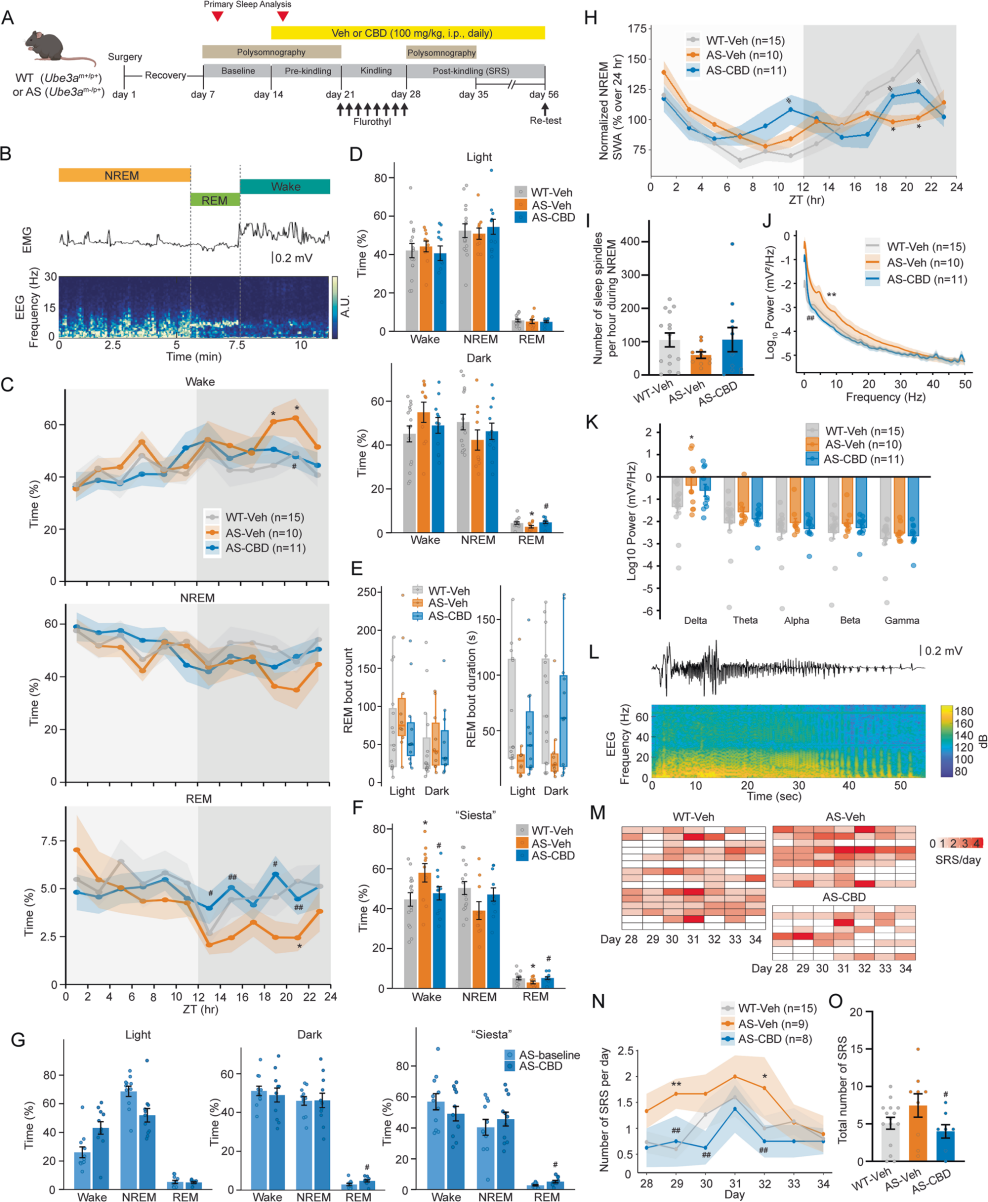
CBD corrects sleep deficits and reduces spontaneous seizures in AS model mice. **A** Schematic of experimental design. **B** Representative vigilance states segmentation based on EMG and EEG. **C** Percent of wake, NREM, and REM time of WT-Veh, AS-Veh, and AS-CBD mice in 2 h bins across 24 ZT. **D** Percent of wake, NREM, and REM time of WT-Veh, AS-Veh, and AS-CBD mice summarized during the light or dark cycle. **E** REM sleep bout count and bout duration during light and dark cycles. **F** Percent of wake, NREM, and REM time of WT-Veh, AS-Veh, and AS-CBD mice during the “Siesta” period (i.e., ZT 18–22). **G** Percent of wake, NREM, and REM time of AS mice before (baseline) and after CBD treatment during the light, dark, and “Siesta” period. **H** Normalized NREM SWA as a percentage of 24 h mean, grouped into WT-Veh, AS-Veh, and AS-CBD. **I** Number of sleep spindles per hour of NREM sleep in WT-Veh, AS-Veh, and AS-CBD mice. **J** Power spectral density (PSD, 0–50 Hz) of WT-Veh, AS-Veh, and AS-CBD mice averaged across 24 ZT. **K** Sum of total powers within each frequency band of delta (0.5–4 Hz), theta (4–8 Hz), alpha (8–12 Hz), beta (12–30 Hz), and gamma (30–70 Hz) from WT-Veh, AS-Veh, and AS-CBD mice. **L** Representative spontaneous electrographic seizure showing initiation, evolution, termination, and postictal suppression accompanied with its spectrogram. **M** Number of spontaneous recurrent seizures (SRS) and **N** their average per day. **O** Total number of SRS over 7 days post flurothyl kindling in WT-Veh, AS-Veh, and AS-CBD mice. Data were presented as (**C, D, F, G, H, I, J, K, N, O**) mean ± standard error or **E** median with interquartile range and analyzed using the Kruskal-Wallis test followed by Dunn’s post hoc comparison (**C, D, E, F, J, K**), Wilcoxon signed-rank test (**G**), or one-way ANOVA followed by post hoc Tukey’s test (**H, I, N, O**). *p < 0.05 and **p < 0.01 compared to WT-Veh. ^#^*p* < 0.05 and ^##^*p* < 0.01 compared to AS-Veh (**C, D, F, H, J, N, O**) or AS-baseline (**G**).

Sleep problems such as sleep/wake rhythm disorders, multiple nocturnal awakenings, or difficulties in falling asleep are common in AS individuals [7, 8]. AS model mice faithfully recapitulate many sleep abnormalities, with reduced REM sleep being one of the most profound and reproducible sleep phenotypes [9–11]. Consistent with previously published results, we observed sleep abnormalities, particularly a significant (*p* < 0.05) reduction in REM sleep time during the dark cycle, in AS-Veh mice (2.75 ± 0.33%) compared with WT-Veh mice (4.61 ± 0.36%) (Fig. 1A–D). Chronic CBD treatment significantly increased (*p* < 0.05) the dark cycle REM sleep time in AS mice (4.82 ± 0.37%), restoring it to WT levels (Fig. 1A–D). Since total REM sleep reflects both bout frequency and bout duration, we next examined these parameters to determine whether the observed changes were driven by alterations in episode number, bout length, or both. We found that the different REM sleep times are likely due to changes in REM bout duration (a 69% reduction in AS-Veh compared to WT-Veh, with a non-significant trend toward correction by CBD, *p* = 0.081), rather than changes in REM bout number (Fig. 1E). Previous studies also showed that AS model mice lack the active-phase rest period (also known as “nocturnal nap” or “Siesta”), which is commonly observed in the WT mice around Zeitgeber Time (ZT) 18–22 [11]. Consistently, we found a notable increase in wakefulness and a decrease in NREM and REM sleep time during ZT 18–22 in AS-Veh mice compared with WT-Veh mice. The loss of “Siesta” in AS mice was corrected by CBD treatment to the levels of WT mice (Fig. 1F). Importantly, CBD treatment also ameliorated the REM sleep deficits of AS model mice during both the dark cycle and “Siesta” period compared to their own baseline recordings before treatment. We further noted a trend toward increased wakefulness (*p* = 0.054) and decreased NREM sleep (*p* = 0.083) during the light cycle after CBD treatment compared to the baseline, which may be attributable to handling and injection during this period (Fig. 1G).

Slow-wave activity (SWA) and sleep spindles are EEG hallmarks of NREM sleep. Previous studies have shown that AS model mice have impaired sleep pressure accumulation, with lower NREM SWA during the late dark cycle and fewer sleep spindles than WT mice [11]. Given this, we first investigated the effects of CBD on sleep homeostasis by measuring NREM SWA delta power. We found that WT-Veh mice exhibited a typical elevation of SWA toward the end of the dark cycle (i.e., ZT 20–24). In contrast, AS-Veh mice showed a slower increase in SWA during the same time frame and thus failed to build sleep pressure. CBD treatment partially restored this sleep-pressure accumulation in AS mice during the late dark cycle by increasing SWA (Fig. 1H). Surprisingly, CBD treatment also moderately increased SWA during the late light cycle, which could result from the acute effects of the daily CBD bolus administered around ZT 4–6 h (Fig. 1H). We next quantified sleep spindles during NREM sleep, adding another clinically relevant functional phenotype for therapeutic evaluation [9]. We found a trend toward reduced sleep spindles in the AS-Veh mice (60 ± 9.7), although the difference was not statistically significant compared with the WT-Veh mice (105.6 ± 20.9, *p* = 0.36). CBD treatment increased sleep spindle counts back to the WT levels (106.4 ± 36.2); however, this difference was not statistically significant (*p* = 0.58) (Fig. 1I). In summary, compared with Veh, CBD improved sleep across multiple facets, including increasing REM sleep time during the dark cycle, restoring the impaired “Siesta” and sleep pressure accumulation during the late dark cycle, as well as a non-significant trend toward correcting sleep spindle deficits in AS model mice.

### CBD partially rescues the brain oscillation impairments in naïve AS model mice

Brain oscillation alterations, particularly the elevated delta power, are reliable biomarkers of AS in both preclinical and clinical settings [12]. Elevated delta power is also associated with cognitive outcomes in AS [13]. We therefore conducted power spectral analysis of EEG data collected from the epidural electrodes. Our previous work demonstrates that acute CBD treatment can mitigate elevated motor cortex local field potential, including both delta and theta (4–8 Hz) activity, in AS model mice [6]. Corroborating these results, we found that chronic CBD treatment attenuated the overall elevation of epidural cortical low-frequency oscillations in AS-Veh compared to WT-Veh mice (Fig. 1J). However, these rescue effects did not reach statistical significance when the delta, theta, alpha (8–12 Hz), beta (12–30 Hz), and gamma (30–70 Hz) frequency bands were analyzed separately after adjustment for multiple comparisons (Fig. 1K). Biologically, this pattern suggests a diffuse modulation of network activity rather than a strong, frequency-specific effect.

### CBD reduces spontaneous recurrent seizure (SRS) frequency in the AS model mice

Our previous work shows the effectiveness of acute CBD treatment in reducing the severity and duration of audiogenic and hyperthermia-induced seizures in AS model mice [6]. However, these paradigms rely on externally evoked events and may not fully capture the therapeutic impact of CBD on the SRS that define epilepsy. Because naïve AS model mice do not exhibit typical SRS with overt behavioral manifestation, we took advantage of an eight-day flurothyl kindling, which is known to elicit a rapid evolution of SRS that remits within weeks, modeling the process of epileptogenesis [14]. We monitored the development of SRS (Fig. 1L) within a one-week time window following the completion of flurothyl kindling to assess the effects of CBD on the occurrence of SRS in kindled AS model mice. We found a rapid increase in the number of SRS in AS-Veh mice compared with WT-Veh mice. Chronic treatment of CBD significantly (*p* < 0.05) reduced the number of SRS in AS mice (Fig. 1M–O). Interestingly, CBD treatment had little effect on the flurothyl-induced seizure threshold during the 8-day kindling or retest (data not shown).

## Discussion

This study primarily suggests that CBD restores REM sleep deficits in AS model mice. One hypothesis is that this effect may be related to CBD’s ability to enhance acetylcholine signaling [15], a central regulator of REM sleep. CBD’s ability to reduce the frequency of SRS, beyond its effects on induced-seizure suppression [6], further strengthens the preclinical evidence supporting its potential for clinical translation. While these results provide a valuable preclinical roadmap for human clinical trials, this study was conducted in adult mice, whereas AS symptoms are often most severe during childhood. Future research is needed to determine if CBD offers similar benefits during early development. We also acknowledge the limitations of excluding the WT-CBD group in the study design because our current design efficiently addresses the key scientific question of CBD’s potential therapeutic effect in AS while minimizing animal use.

## Supplementary information

Supplemental Material

## Data Availability

The data that support the findings of this study are available from the corresponding author on reasonable request.
