# Supplementary material for: Cannabidiol corrects sleep deficits and reduces spontaneous seizures in Angelman syndrome model mice
Source: Neuropsychopharmacology. 2026 Jun 9;51(9):1737–40. doi: 10.1038/s41386-026-02462-7 (PMC13389062; doi:10.1038/s41386-026-02462-7)
Supplement: Supplementary file 1 — Supplemental Material [file 41386_2026_2462_MOESM1_ESM.docx]

**Supplementary Materials for**

**Cannabidiol Corrects Sleep Deficits and Reduces Spontaneous Seizures in Angelman Syndrome Model Mice**

Tyler Shannon^1^, Mariam Najeeb^1,2^, Yoon-Jae Yi^1^, Yuyan Shen^1,3^, and Bin Gu^1*^

^1^Department of Neuroscience, The Ohio State University, Columbus, USA

^2^Neuroscience Program, The Ohio State University, Columbus, USA

^3^College of Veterinary Medicine, The Ohio State University, Columbus, USA

*Corresponding author:

Bin Gu, Ph.D.

460 W 12th Avenue, 612 Biomedical Research Tower, Columbus, OH 43210, USA

[bin.gu@osumc.edu](mailto:bin.gu@osumc.edu)

**The supplementary materials include:**

**Supplemental Methods**

**Supplemental Methods**

**Mice**: Adult (2-4 months) AS model mice (B6.129S7-*Ube3a^tm1Alb^*/J, Jackson Laboratory #016590) and their littermate wildtype (WT) controls were randomly divided into three treatment groups: WT mice (*Ube3a^m+/p+^*) treated with vehicle (WT-Veh); AS model mice (*Ube3a^m–/p+^*) treated with vehicle (AS-Veh); and AS model mice (*Ube3a^m–/p+^*) treated with CBD (AS-CBD). Male and female mice were distributed evenly across all experimental groups. No significant sex differences (p > 0.05) were detected in any measured outcomes; therefore, males and females were pooled for subsequent analyses. Our primary objective was to assess the therapeutic effects of CBD in AS. This design efficiently addressed the key scientific question while minimizing animal use. All animal procedures were approved by the Institutional Animal Care and Use Committee of the Ohio State University and performed in accordance with the guidelines of the U.S. National Institutes of Health. Mice were group-housed before surgery on a 12:12 light/dark cycle.

**Treatment:** Epidiolex^®^ (CBD) and placebo control (Veh) were provided by Jazz Pharmaceuticals. The composition of the vehicle includes: 79 mg/mL of anhydrous ethanol, 0.5 mg/mL of sucralose, 0.2 mg/mL of strawberry flavor, and up to 0.1 mg/mL of beta carotene in refined sesame oil. CBD was prepared in the vehicle at a concentration of 20 mg/mL. We aliquoted them into 1 mL and stored them at room temperature, away from light, until use. We injected Veh or CBD (100 mg/kg, 0.1 mL per 20 g body weight) via intraperitoneal (i.p.) injection. During flurothyl kindling, vehicle and CBD were administered one hour before each flurothyl exposure.

**Surgery:** Mice were anesthetized using isoflurane via compressed room air. Ethiqa (3.25 mg/kg, s.c. single dose before surgery) and Ibuprofen (40mg/kg, via drinking water starting 24 hr prior to surgery and continued for 7 days post injury) were administered for analgesia. The mouse was placed in the stereotactic apparatus, and a midline scalp incision was made. Three Burr holes were drilled into the skull, the first one over the frontal cortical area (1 mm anterior to bregma, 1.5 mm lateral to the midline) and the second one over the parietal area (1 mm anterior to lambda, 1.5 mm lateral to the midline) of the right hemisphere for EEG recordings, and the third one above cerebellum (1 mm posterior to lambda, -1 mm lateral to the midline) for ground electrode. Stainless steel EEG recording screws (1.0 mm diameter) that are connected to male pins via silver wires (0.25 mm diameter) were placed in the holes for epidural positioning over the cortex. We punched small bilateral holes in the trapezius muscles and inserted the insulated stainless steel wires with ~1mm bare ends that serve as EMG electrodes. All wires connected to male pins were mounted on a mini 6-channel pedestal. The entire electrode assembly was fixed to the skull using dental cement.

**Experimental design:** Mice were acclimated during recovery for 7 days after surgery before polysomnography recordings in the same room with automatically controlled 12-hour light/12-hour dark cycles (lights on at 06:00). Mice were individually housed and recorded 24/7 during three one-week recording sessions: baseline (day7–day14), pre-kindling (day7–day21), and post-kindling (day28–day35). Flurothyl kindling was conducted during day21–day28 followed by a flurothyl re-test on day56. Mouse remained in the same room during day21–day28 and day35–day56 when the polysomnography was not conducted. Mice received daily vehicle or CBD injection from day14 to day56 as detailed in the treatment plan.

**Chronic video-EEG-EMG recording:** A tethered system with a commutator was used to allow each mouse to roam freely within the home cage and enable time-locked video-EEG-EMG polysomnography recording. EEG recordings were sampled at 2000 Hz and filtered with 0.3 Hz high-pass and 1000 Hz low-pass filters. EMG recordings were sampled at 1000 Hz and filtered with 10 Hz high-pass and 70 Hz low-pass filters. A 60 Hz notch filter was applied. Ponemah 5.3 software was used for signal streaming, raw data acquisition, and video (infrared-enabled for night vision) synchronization.

**Sleep analyses**: We used an open-source rodent sleep state scoring tool, AccuSleep, for automated and unbiased sleep analysis. For each mouse, we trained Accusleep at a 2.5-s epoch resolution and assigned it to one of three stages based on patterns of EEG and EMG activity. Vigilance states were classified as awake (low-voltage, high-frequency EEG; high-amplitude EMG), REM sleep [low-voltage EEG with a predominance of theta activity (6–10 Hz); very low amplitude EMG], or NREM sleep (high-voltage, mixed-frequency EEG; low-amplitude EMG). Sleep spindles were analyzed using the sleep spindle detection function through Yet Another Spindle Algorithm (YASA), an open source Python toolbox. Sleep spindles are defined by EEG waves within the 11–16 Hz frequency range, with a duration of greater than 0.5 s. Slow wave activity measured as spectral power in the 0.5–4 Hz frequency range exclusively during NREM sleep, and expressed as a percentage of total spectral power in the EEG signal (0.5–100 Hz) during that time period. Primary sleep analyses were restricted to a 24-hour epoch, second day of dosing (day16), chosen a priori to capture effects after stabilization of repeated CBD exposure, when plateau plasma levels were achieved. A corresponding pre-treatment time window (day 9) was analyzed as the baseline for sleep. This strategy reduces variability from the initial handling/novelty response and avoids longer-term multi-day confounds, while providing a consistent and representative measure across animals.

**Power spectral analysis**: The power spectral density was analyzed using customized Python algorithms. We averaged the power spectral density over 24 hr recordings to examine overall alterations in brain oscillations across 12 hr light and 12 hr dark cycles. For the power spectrum, the EEG data were first transformed using a Morlet Wavelet with a 3s time resolution and a 0.5 Hz step size. Then the data were further divided into particular wave bands for band-specific quantification: delta (0.5–4 Hz), theta (4–8 Hz), alpha (8–12 Hz), beta (12–30 Hz), and gamma (30–70 Hz).

**Flurothyl kindling:** For flurothyl-induced seizures, each mouse was habituated for 1 min in a 2-liter glass chamber before the top of the chamber was closed. Then, 10% flurothyl (bis-2,2,2-trifluoroethyl ether) in 95% ethanol was infused at a rate of 200 µL/min onto a disk of filter paper suspended at the top of the chamber. Upon the emergence of a generalized seizure, the chamber lid was immediately removed, allowing rapid dissipation of the flurothyl vapors and exposing the mouse to fresh air. Mice were returned to their home cage following recovery from behavioral seizures. For kindling, flurothyl exposures were repeated once daily over eight consecutive days (induction phase). After completion of the induction phase, mice were given a 28-day rest period (incubation phase), during which they remained in their home cages without flurothyl exposure, and were then re-exposed to flurothyl on day 36 (retest). Mouse behavior during each flurothyl exposure was recorded on video.

**Spontaneous seizure analyses**: For the detection of spontaneous recurrent seizures, the raw EEG data were converted into .edf before being imported into Sirenia Seizure Pro (Pinnacle Technology, Inc.). The automatic seizure detection function of Sirenia Seizure Pro was applied. All possible positive seizure episodes identified using the automatic seizure detection function were manually reviewed and validated by investigators who were blind to genotype and treatment. A seizure is defined as an episode of rhythmic EEG discharges (with an amplitude greater than twice the baseline, lasting more than 5 s) that exhibit clear initiation, evolution, and termination. Concurrent behavioral seizure manifestations were also confirmed.

**Rigors and statistics:** All experiments were conducted by investigators blinded to genotype and treatment. Data from male and female mice (no significant difference) were combined for inter-group analysis. All statistical analyses were performed using appropriate R libraries. The Shapiro-Wilk normality test was performed to justify the use of parametric statistical tests. If the data failed the normality test, we applied appropriate nonparametric tests instead. Depending on the normality, data were analyzed using Kruskal-Wallis test followed by Dunn’s post hoc comparison or Wilcoxon signed-rank test for nonparametric data and one-way ANOVA followed by post hoc Tukey’s test for parametric data.
